# Chloride Intracellular Channel 2 Can Function as a Malignant Factor in Head and Neck Squamous Cell Carcinoma

**DOI:** 10.1002/hed.70133

**Published:** 2025-12-12

**Authors:** Yuki Hosokawa, Shoko Miyoshi, Yuji Hayashi, Yuki Irifune, Eriko Sato, Sohei Mitani, Mohammed Choudhury, Hajime Yano, Junya Tanaka, Naohito Hato

**Affiliations:** ^1^ Department of Otorhinolaryngology, Head and Neck Surgery, Graduate School of Medicine Ehime University Matsuyama Ehime Japan; ^2^ Department of Molecular and Cellular Physiology, Graduate School of Medicine Ehime University Matsuyama Ehime Japan; ^3^ Department of Otorhinolaryngology, Head and Neck Surgery Ehime Prefectural Central Hospital Matsuyama Ehime Japan; ^4^ Department of Anesthesiology and Perioperative Medicine, Graduate School of Medicine Ehime University Matsuyama Ehime Japan

**Keywords:** chloride intracellular channel 2 (CLIC2), head and neck squamous cell carcinoma (HNSCC), malignant potential, risk factor, therapeutic target

## Abstract

**Background:**

The association of chloride intracellular channel 2 (CLIC2) with tumors has remained unclear. However, recently, CLIC2 was found to exhibit antitumor properties in some tumors. In this study, we aimed to evaluate the clinical relevance of CLIC2 expression in head and neck squamous cell carcinoma (HNSCC) to determine whether it exerts antitumor effects similar to those in brain tumors and to explore its potential as a novel therapeutic target in HNSCC.

**Methods:**

We explored the significance of CLIC in HNSCC using cell biological analyses and investigated the gene expression profile of the CLIC2 forced‐expressed HNSCC cell line. CLIC2 expression in human HNSCC was examined histopathologically.

**Results:**

Forced expression of CLIC2 in HNSCC cells was accompanied by increased cell proliferation, resistance against natural killer cells, and expression of tumor‐promoting genes in addition to increased tumorigenicity upon xenografting in a mouse model. CLIC2 expression was observed in several human cases of HNSCC.

**Conclusion:**

CLIC2 may act potentially as a novel risk factor for HNSCC.

**Abbreviations**
CGP: comprehensive genomic profiling
CLIC2: chloride intercellular channel 2
HNSCC: head and neck squamous cell carcinoma
ICIs: immune checkpoint inhibitors
MMP: matrix metallopeptidase
NK: natural killer

## 1 Introduction

Head and neck squamous cell carcinoma (HNSCC) accounts for 4.5% of all new cases of cancer in the Japanese population. It remains clinically important owing to its frequent association with functional impairments, including dysphagia, voice impairment, and respiratory dysfunction, resulting from disease progression or treatment, with quality‐of‐life management being a major concern. The incidence and mortality of HNSCC have increased in recent years [1, 2, 3, 4]. Platinum‐based chemotherapy with cisplatin has long been the standard of care; however, several concerns, including high recurrence rates and a significant physical burden following adverse events, persist [2, 5]. The development of immune checkpoint inhibitors (ICIs) and photoimmunotherapy has raised the possibility of prolonged survival; nevertheless, cisplatin remains the key drug for HNSCC. Furthermore, the prognoses for patients refractory or intolerant to platinum‐based chemotherapy remain poor [5, 6, 7]. The widespread implementation of comprehensive genomic profiling (CGP) in certain tumor types has increased the opportunities for identifying recommended therapeutic agents. However, such opportunities remain limited in HNSCC, and the cases in which the identified targets lead to treatment are extremely rare [8, 9]. This highlights the need for discovering novel therapeutic targets for HNSCC.

Chloride intracellular channel proteins (CLICs) are small globular proteins that have been identified as chloride ion channels with a molecular mass of approximately 30 kDa, comprising six members—CLIC 1–6 [10, 11]. However, CLICs are rarely localized to the plasma membrane as transmembrane proteins despite their designation as “channels” and are found predominantly in the soluble fractions of the cytosol and nuclei [11, 12]. CLICs are suggested to play various roles beyond their function as ion channels, including ryanodine receptor modulation (RyRs) [13], intracellular trafficking [14], and tumor growth factor‐β (TGFβ)‐mediated signal modification [15]. However, recent database analyses have revealed that the expression levels of CLICs may affect cancer prognosis [11, 12, 16]. The association between CLICs expression and tumors varies based on the tumor type [16, 17]. Notably, CLIC4 has been studied extensively, and its high expression is associated with poor prognosis in many tumor types [12, 18, 19]. In contrast, CLIC2 is expressed in the endothelial cells of normal blood vessels; however, it shows extremely low expression in malignant cells, and its association with cancer remains the least studied among the CLIC family [12, 16]. Recent in silico analyses have shown that high CLIC2 expression correlates with prolonged survival in lung, gastric, breast, and liver cancers, indicating a potential anticancer effect [12, 20, 21]. Using a brain tumor model, we have previously demonstrated that CLIC2 suppresses invasion and metastasis by inhibiting matrix metallopeptidase 14 (MMP14) activity, proposing its function as a cancer‐suppressive molecule, at least in brain tumors [16, 22].

MMPs are enzymes that function in tissue remodeling under physiological conditions. It is widely known that they can be “abused” in cancer progression and malignant transformation [23]. Thus, the finding that CLIC2 can suppress MMP14 in glioma cells has provided hope for the development of new therapies. Since the role of MMPs in cancer progression and malignant transformation has been reported to be similar in HNSCC [24, 25], it will be interesting to determine whether the inhibitory regulation of CLIC2 on MMPs is similar in HNSCC.

On the other hand, in the process of analyzing the metastatic potential of HNSCC cells, we found that simultaneous inhibition of two factors, Na^+^/H^+^ exchange transporter 1 (NHE1) and lysyl oxidase‐like factor 2 (LOXL2), makes HNSCC cells susceptible to elimination by natural killer (NK) cells [26]. The association with these factors may also be a consideration in evaluating the significance of CLIC2 in HNSCC.

However, CLIC2's role in HNSCC remains unclear because of the lack of CLIC2 studies in the HNSCC field. In this study, we aimed to evaluate the clinical relevance of CLIC2 expression in HNSCC to determine whether CLIC2 exerts anticancer effects similar to those observed in brain tumors. Furthermore, we aimed to explore its novel therapeutic target potential in HNSCC.

## 2 Materials and Methods

### 2.1 Cells

The human tongue squamous cell carcinoma cell line SAS and its metastatic derivative SASL1m, which were previously established to construct a murine metastasis model, were used in this study [27]. These cells were maintained in Dulbecco's Modified Eagle Medium (FUJIFILM Wako, Tokyo) supplemented with 10% fetal calf serum (Nichirei, Tokyo) and antibiotics (penicillin–streptomycin–amphotericin B suspension (×100); FUJIFILM Wako, Tokyo) [26, 28]. For NK cells, the NK92MI cell line was used. The cell line was maintained in the KBM501 medium for human lymphocyte activation (Kohjin Bio, Saitama, Japan).

### 2.2 Animal Experiments

All animal experiments were performed in accordance with the guidelines of the Ethics Committee for Animal Experimentation at Ehime University (approval number: 05‐U‐44‐16). KSN nude mice were subjected to xenograft experiments as described below.

### 2.3 Xenograft

KSN nude mice aged 8–12 weeks were utilized in this study. The tumor cells were suspended in phosphate‐buffered saline (PBS) at 1 × 10^6^ cells/50 μL and injected subcutaneously into the dorsal flank using a 27 Gauge needle of an insulin syringe under isoflurane anesthesia. Mice with failed or leaked injections due to skin penetration were excluded from the analysis. The mice were euthanized, and subcutaneous tumors were harvested 5 weeks after the xenograft. Tumor volume was assessed by measuring the long and short axes and the thickness.

### 2.4 Immunoblotting

SAS and SASL1m cells were lysed in 2% sodium dodecyl sulfate (SDS) to extract proteins. Lysates from a benign human meningioma, reported by Ozaki et al., were used as the reference sample [22]. Protein quantification, SDS‐polyacrylamide gel electrophoresis (PAGE), transfer, antibody reaction, and detection were all performed according to previously described methods [28]. Samples were subjected to SDS‐PAGE at 10 μg/lane. The antibodies used in this study are listed in Table S1.

### 2.5 Quantitative Real‐Time RT‐PCR (qPCR)

qPCR was performed according to previously published protocols [29]. The primers used are listed in Table S2. The mRNA expression values were presented as relative expression levels normalized to glyceraldehyde‐3‐phosphate dehydrogenase (*GAPDH*).

### 2.6 Establishment of a Forced CLIC2‐Expressing HNSCC Cell Line

A cell line stably expressing CLIC2 was established using SASL1m, according to a method described previously [22, 30]. Furthermore, SASL1m cells transfected with an empty pCX4 vector were established as negative controls (nc).

### 2.7 Cell Growth Curve

On Day 0, each tumor cell line at 1 × 10^4^ cells per dish was inoculated in five 3.5 cm dishes. One dish per day was stained with Hoechst 33342 to visualize the nuclei from Days 1 to 5, and images were captured from five fields per dish. The nuclei were counted using the ImageJ software (National Institutes of Health, Bethesda, MD, USA), and the increase in cell number was measured every 24 h. Three independent experiments were conducted, and statistical analyses were performed.

### 2.8 Co‐Culture Assay

Each tumor cell line was inoculated on Day 0 at 1.5 × 10^5^ cells per dish in three 3.5 cm dishes. On Day 1, the culture medium was replaced with NK92MI medium, and 3 × 10^4^ NK92MI cells were overlaid on each dish. The floating cells were washed with PBS after 24 h of incubation (Day 2), and the remaining adherent cells were stained with Hoechst 33342. Five microscopic fields per dish were imaged, and the ImageJ software was used to measure the number of nuclei. Subsequently, statistical analysis was performed.

### 2.9 Next‐Generation Sequencing (NGS) Analysis

The preparation of the mRNA library was outsourced to Nippon Genetics (Tokyo), who prepared it using the NEBNext Ultra II RNA Library Prep Kit for Illumina. Sequencing was performed using the MGI DNBSEQ T7 platform. Adapter trimming was performed with Trim Galore, and quality control was assessed using FastQC. RNA reads were mapped to the human reference genome (GRCh38) using HISAT2, and gene expression levels were quantified using StringTie. Differentially expressed gene (DEG) analysis was conducted using edgeR in the TCC‐GUI using TNM normalization. The following lists of genes were used for the enrichment analysis: (1) a composite list of the top 50 up‐ and downregulated genes, respectively, (2) a list of significantly upregulated genes (*p* < 0.05), and (3) a list of significantly downregulated genes (*p* < 0.05).

### 2.10 Immunohistochemistry

The preparation and use of paraffin blocks from HNSCC surgical specimens was approved by the Clinical Research Ethics Committee of Ehime University Hospital (approval number: 2309004). Clinical observations of the HNSCC surgical specimens examined are summarized in Table 1. Briefly, antigen retrieval was performed using a pressure cooker with citrate buffer (pH 6.0) at 95°C for 5 min after sectioning and deparaffinization. The sections were permeabilized with Tris‐buffered saline with Tween 20 for 15 min, endogenous peroxidase activity was quenched with 1% hydrogen peroxide for 10 min, and non‐specific binding was blocked using the ImmunoCruz rabbit ABC Staining System (sc‐2018; Santa Cruz Biotechnology Inc., TX, USA) for 60 min. Primary antibody reaction was performed overnight at 4°C using an anti‐CLIC2 antibody (1:100) as listed in Table S1. Sections not treated with the primary antibody were used as nc in serially prepared sections. Furthermore, the sections were incubated with a biotin‐labeled secondary antibody for 90 min according to the instructions of the ABC kit, followed by a 30‐min incubation with AB enzyme reagent following the primary antibody incubation. The peroxidase reaction was visualized by incubation with a peroxidase substrate for approximately 5 min. Subsequently, hematoxylin counterstaining was performed after rinsing with distilled water, and the slides were mounted. The stained sections were observed and photographed under a microscope (OLYMPUS IX‐71, Tokyo, Japan).

**TABLE 1:** Summary of clinical observations for immunohistochemical analyses in Figure 7. Gender, age, and each clinical observation of the specimen were summarized.

Age	Sex	Organ	Pathology diagnosis	pT	pN	pM	pStage	CLIC2‐expression	Recurrence or metastasis
76	M	Hypopharynx	SqCC	2	0	0	II	+	Lymph node
74	M	Larynx	SqCC	2	0	0	II	−	No recurrence
89	M	Oropharynx	SqCC	2	0	0	II	−	No recurrence
79	M	Larynx	SqCC	3	0	0	III	−	Lung
64	M	Larynx	SqCC	3	0	0	III	−	No recurrence
88	M	Oropharynx	SqCC	3	0	0	III	−	No recurrence
69	M	Hypopharynx	SqCC	2	1	0	III	−	No recurrence
57	M	Oropharynx	SqCC	2	1	0	III	+^b^	Local
68	M	Tongue	SqCC	4a	0	0	IVA	+	No recurrence
67	F	Tongue	SqCC	4a	0	0	IVA	−	Local
64	M	Hypopharynx	SqCC	4a	2b	0	IVA	+^a^	Lymph node
66	M	Hypopharynx	SqCC	3	3b	0	IVB	+	No recurrence
61	M	Oropharynx	SqCC	1	3b	0	IVB	+	Lymph node and lung
74	M	Larynx	SqCC	3	3b	0	IVB	+	Lymph node and lung
76	M	Tongue	SqCC	4a	3b	0	IVB	−	No recurrence

^a^ Patient 1 in Figure 7.

^b^ Patient 2 in Figure 7.

### 2.11 Statistical Analysis

Data are presented as mean ± standard error of the mean or standard deviation (SD). Statistical analyses were performed using the Prism 9 software (GraphPad Software, La Jolla, CA, USA). Student's *t* test was used to analyze comparisons between control and CLIC2‐overexpressing cells, except for the cell growth assay, which was performed using multiple *t* tests.

## 3 Results

### 3.1 Establishment of a Forced CLIC2‐Expressed HNSCC Cell Line

**FIGURE 1:**
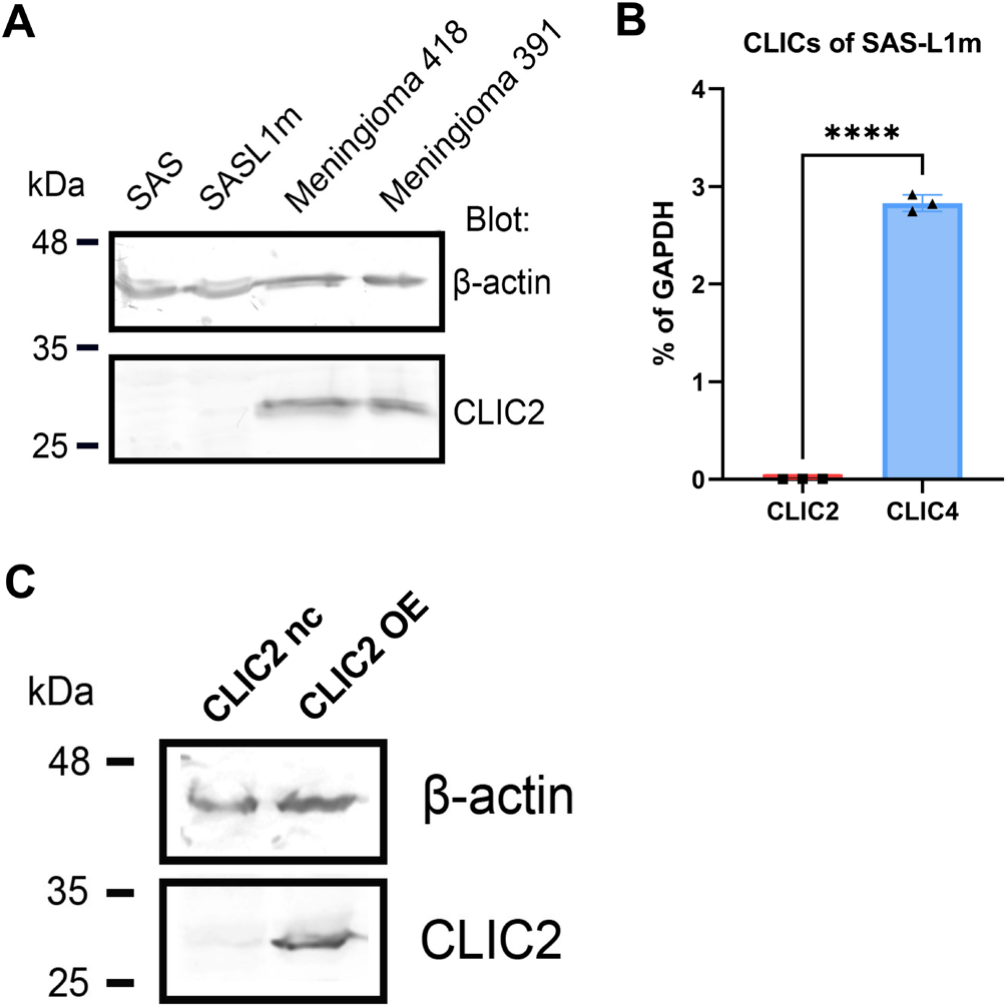
Establishment of HNSCC cells with constitutive CLIC2 overexpression (CLIC2 OE). (A) Immunoblotting analysis of protein levels of β‐actin and CLIC2 in cell lysates obtained by solubilization and recovery with 2% SDS from human HNSCC cell lines (SAS, SAS‐L1m) and meningioma samples (meningioma 418, 391). Each lane was loaded with 10 μg of total protein lysate for the analysis. The antibodies used in this study are listed in Table S1. (B) qPCR analysis of *CLIC2* and *CLIC4* mRNA levels in comparison to those of *GAPDH* in RNA extracted from SAS‐L1m cell line. Primers used are listed in Table S2. **** *p* < 0.0001 (compared with CLIC4 mRNA levels). (C) Immunoblotting analysis of CLIC2 expression in CLIC2 OE and negative control cells (CLIC2 nc). The method used was the same as that for (A). [Color figure can be viewed at wileyonlinelibrary.com]

A subline that constantly expressed CLIC2 in HNSCC cells was generated to explore the significance of CLIC2 in HNSCC. SASL1m cells are a highly metastatic subline of the SAS cell line established from human tongue cancer tissue and recovered from metastases in a mouse lymph node model that underwent metastasis [27]. As far as we examined, CLIC2 expression in malignant cells has been low, so we expected the same to be true for SASL1m. The level of CLIC2 protein in this cell line was compared with that in meningioma cells, in which CLIC2 expression was previously confirmed [22]. Indeed, the expression of CLIC2 was below the detection limit (Figure 1A). Moreover, qPCR analysis revealed that its expression was regulated at the transcriptional level (Figure 1B). The *CLIC2* mRNA level was almost null in comparison to the *GAPDH* mRNA, for example, as compared to that of approximately 3% shown by *CLIC4* (a member of the CLIC family that has been extensively studied in relation to cancer) [18, 19, 31]. Permanent forced expression of CLIC2 in SASL1m cells was achieved by stable transfection of the pCX4 vector bearing *CLIC2* cDNA [22]. The cells of the SASL1m will be referred to as SASL1m CLIC2 overexpression (OE) as CLIC2 was overexpressed in this SASL1m subline (Figure 1C). Furthermore, SASL1m cells transfected with the pCX4 vector without cDNA will be called SASL1m CLIC2 nc (Figure 1C).

### 3.2 Malignant Changes in SASL1m Cell Traits Accompanied CLIC2 OE

**FIGURE 2:**
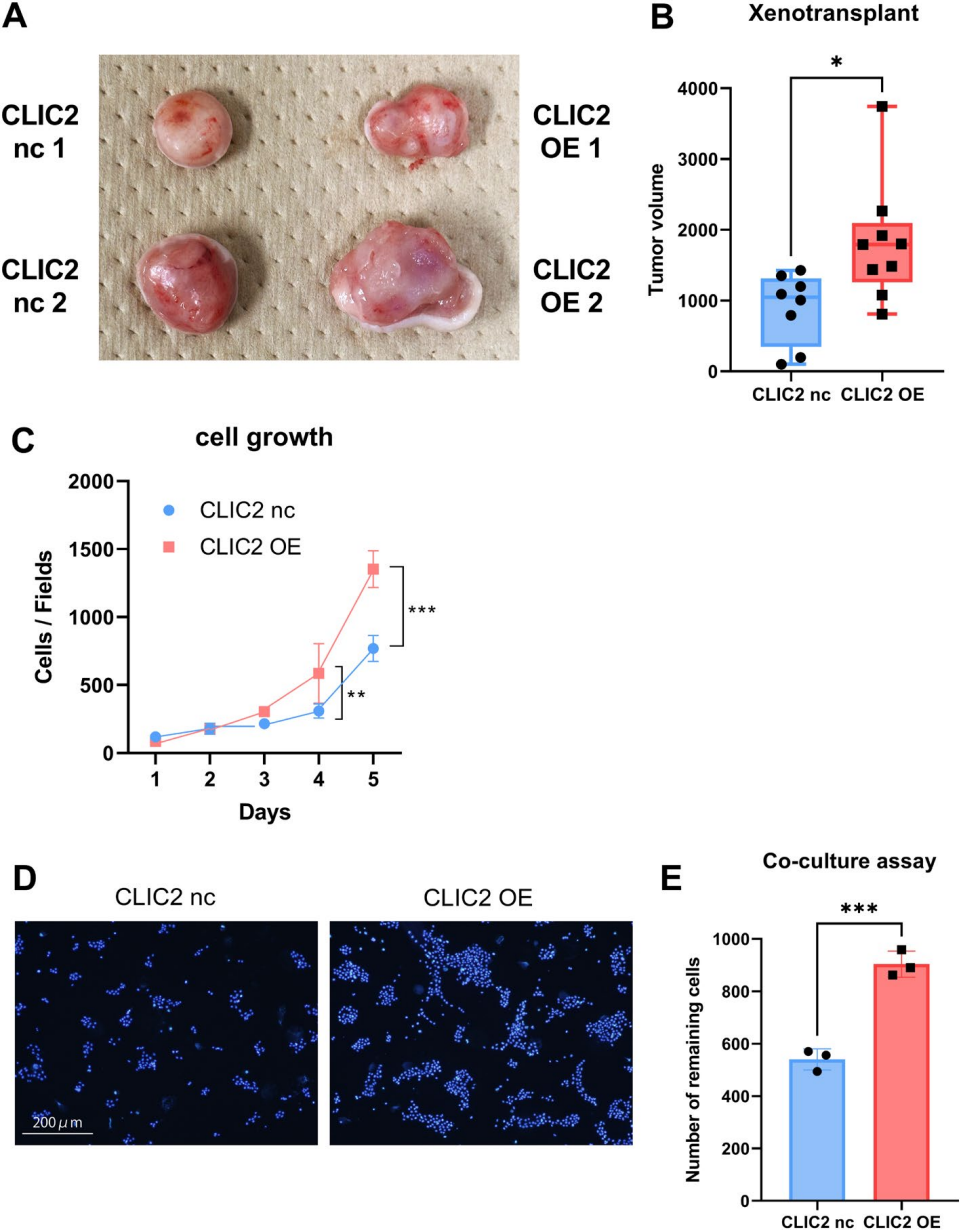
Effects accompanied by CLIC2 overexpression in SAS‐L1m cells. (A) Comparison of primary tumor formation in xenograft models transplanted with CLIC2 nc and CLIC2 OE cells. Primary xenograft lesions were resected 35 days after xenograft implantation into KSN nude mice, as described in Section 2. Eight CLIC2 nc and nine CLIC2 OE mice were used for the analysis. (B) Comparison of the primary xenograft lesion volumes. The tumor volumes measured according to the methods were compared between the CLIC2 nc and CLIC2 OE groups. *p < 0.05 (compared to CLIC2 nc cells). (C) Growth curves. Established cells were seeded at 1 × 10^4^ cells per *φ*3.5 cm dish. The cell numbers were counted every 24 h according to previously described methods, and changes in the number of cells per field were plotted over time. **p < 0.01, ***p < 0.001 (compared with CLIC2 nc cells). (D) Tumor cell elimination by natural killer (NK) cells against CLIC2‐overexpressing cells. Co‐culture of established tumor (CLIC2 nc or CLIC2 OE) and NK cells was performed according to previously described methods. Residual adherent cells were visualized by staining the nuclei with Hoechst 33342. Scale bar: 200 μm. (E) The number of residual adherent cells was quantified from the image data in Figure 3D according to the methods described, and the values were compared between CLIC2 nc and CLIC2 OE cells. ***p < 0.001 (compared with CLIC2 nc cells). [Color figure can be viewed at wileyonlinelibrary.com]

The volume of the xenografted primary tumors formed by CLIC2 OE cell transplantation was larger than that of the nc cells (Figure 2A,B). Therefore, their proliferative potential in culture was measured to investigate which trait changes in the cells might be responsible for the size difference. An increased growth rate was observed with OE cells (Figure 2C). In addition, the cells were co‐cultured with NK92MI [26] to examine the CLIC2 OE cell viability as a reaction to the immune response of the xenograft host. The number of adherent cells after elimination by NK cells in the co‐culture was measured as the number of remaining surviving cells due to SASL1m adherence [26]. OE cells were more persistent than nc cells, and we observed their resistance to NK cells (Figure 2D,E). These results implicated that CLIC2 OE was aggravated, at least in SASL1m cells.

### 3.3 Exploration of Factors Contributing to Tumor Trait Exacerbation Accompanying CLIC2 OE

**FIGURE 3:**
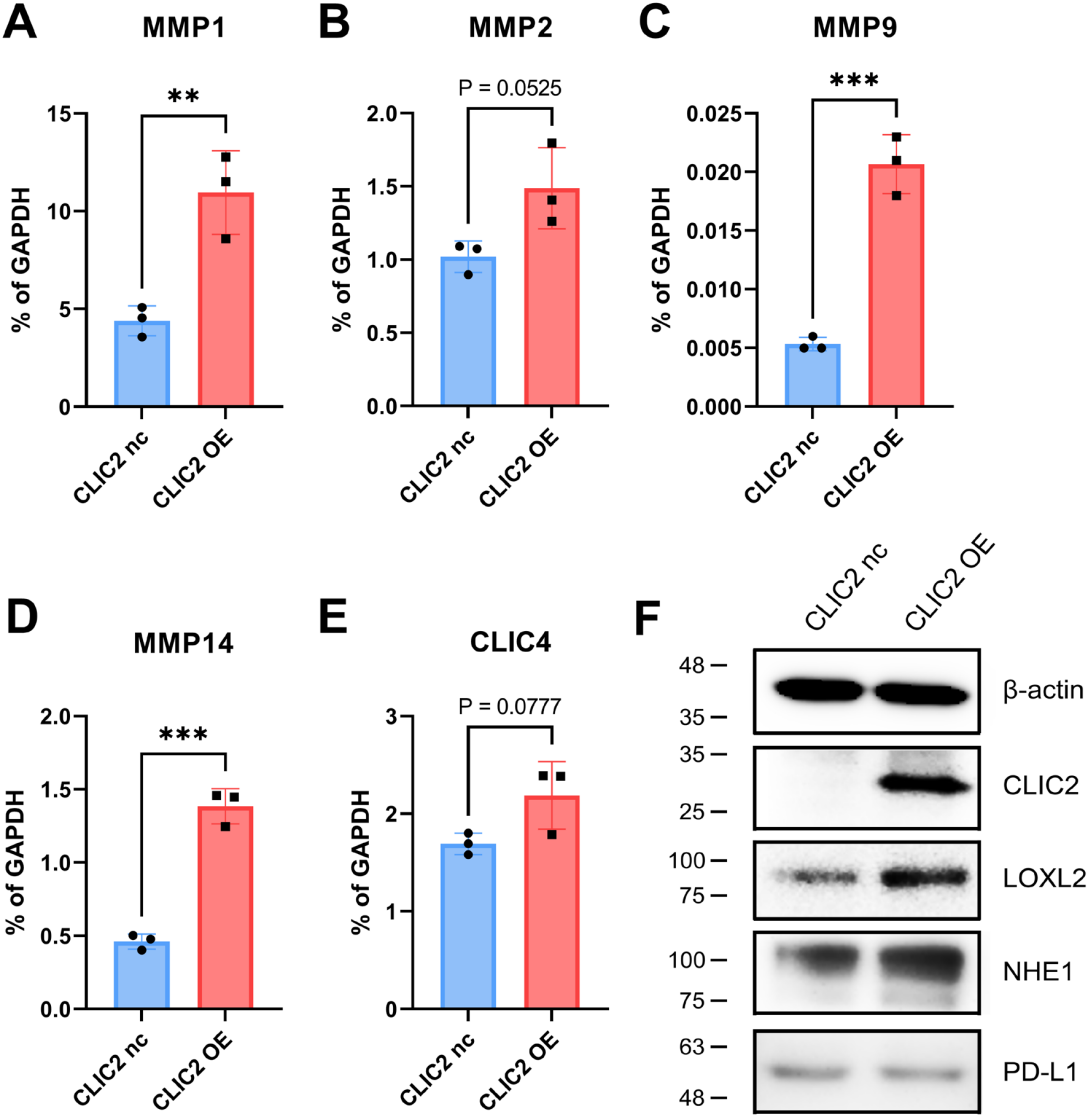
Investigation of the factors involved in the acquisition of a malignant phenotype accompanied by CLIC2 overexpression. (A–E) qPCR analysis was performed on established cells to examine the expression of mRNA previously reported to be altered upon CLIC2 overexpression in glioma cells. **p < 0.01, ***p < 0.001 (compared with CLIC2 nc cells). (F) Immunoblotting was performed in established cells to examine the protein expression changes previously reported to be associated with the malignant phenotypes of HNSCC. [Color figure can be viewed at wileyonlinelibrary.com]

We examined the changes caused by CLIC2 OE in HNSCC that led to tumor trait exacerbation, characteristics of factors associated with CLIC2 in glioma cells, and characteristics of factors associated with cancer progression in SASL1m. In our previous report, we have shown that CLIC2 functions as an antitumor molecule by regulating the matrix metalloprotease family [22]. The mRNA expression of *MMP 1*, *2*, *9*, and *14* increased in SASL1m cells, unlike that observed in glioma cells (Figure 3A–E). Moreover, the protein levels of NHE1 and LOXL2, whose elevated expression was associated with an increased malignancy such as lymph node metastatic potential of SASL1m cells [28, 32], increased with CLIC2 OE (Figure 3F). In contrast, the protein level of the immune checkpoint factor PD‐L1, which has been reported to decrease alongside the suppression of NHE1 function [26, 33] hardly changed upon CLIC2 expression (Figure 3F). These results support that CLIC2 OE is tumor‐promoting, unlike the antitumor effects observed in gliomas.

### 3.4 Genome‐Wide Search for Gene Expression Changes Associated With CLIC2 OE

**FIGURE 4:**
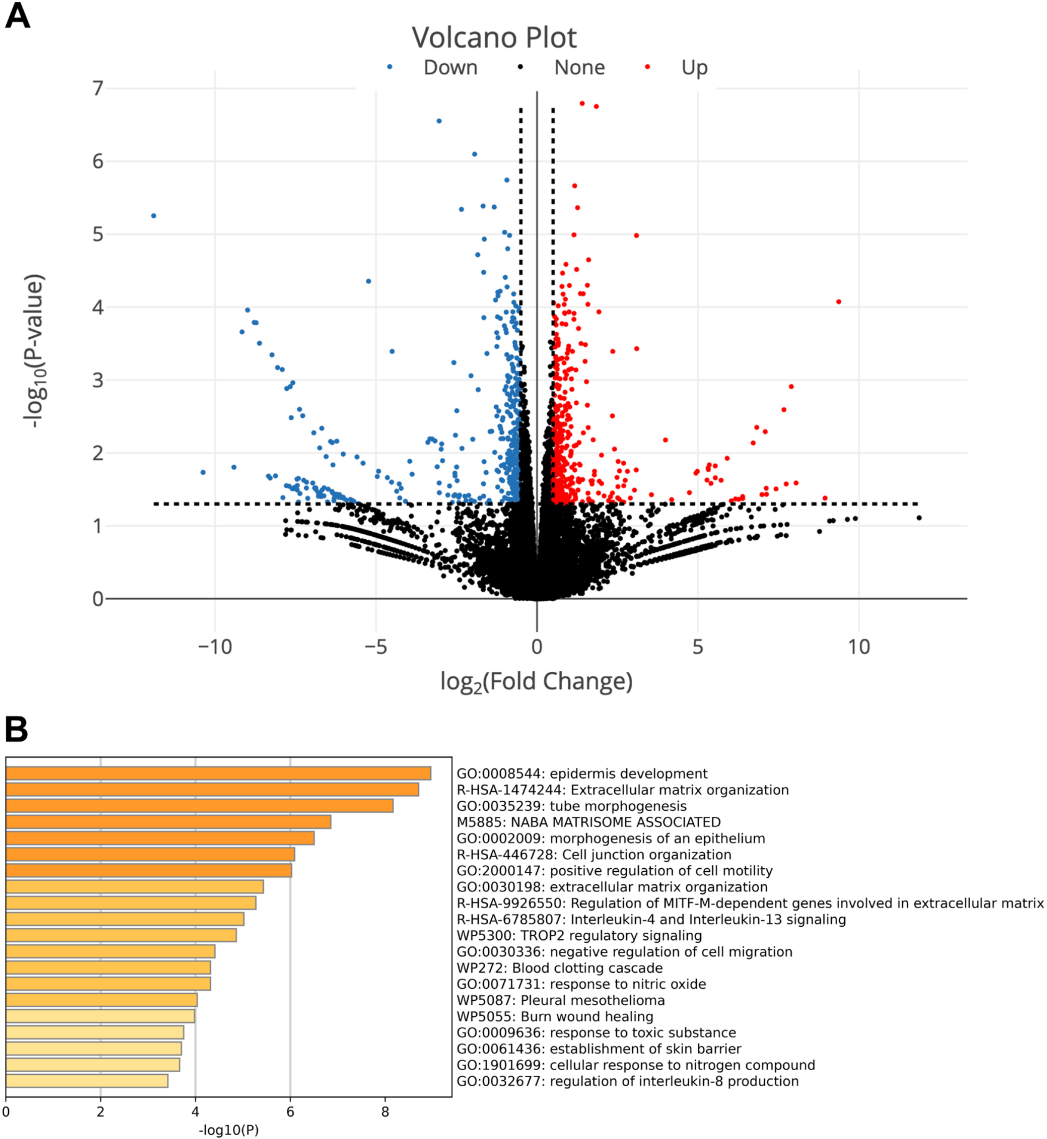
Comprehensive analysis of differentially expressed genes accompanied by CLIC2 overexpression. (A) Volcano plot of the differentially expressed mRNAs associated with CLIC2 overexpression. Red and blue plots indicate the up‐ and downregulated genes, respectively. The analytical method is described in Section 2. (B) Enrichment analysis of differentially expressed genes associated with CLIC2 overexpression. The top 50 upregulated and downregulated genes, respectively (*p* < 0.05) upon CLIC2 overexpression were selected and subjected to Metascape analysis. The top 20 functional terms most strongly associated with the input gene list are shown. They were ranked and color‐coded according to *p* values. [Color figure can be viewed at wileyonlinelibrary.com]

We sought to determine what factors other than those listed in Figure 3 are involved in HNSCC, given that CLIC2 expression is pro‐cancerous in HNSCC. We explored the changes in gene expression associated with CLIC2 OE using mRNA sequencing to comprehensively address this issue. Various genes showed characteristic expression changes (Figure 4A); however, Metascape enrichment analysis revealed changes in factors belonging to pathways, such as “epidermis development,” “extracellular matrix organization,” “tube morphogenesis,” “morphogenesis of an epithelium,” “cell junction organization,” “positive regulation of cell motility regulation,” and others. This indicated that CLIC2 expression may exacerbate the tumor by being involved in these cellular processes (Figure 4B). Further, the results were closely examined considering the results of the mouse model and cell culture experiments, as follows.

### 3.5 Exploration of Cellular Regulatory Pathways in Response to CLIC2 OE, Related to Upregulated Genes

**FIGURE 5:**
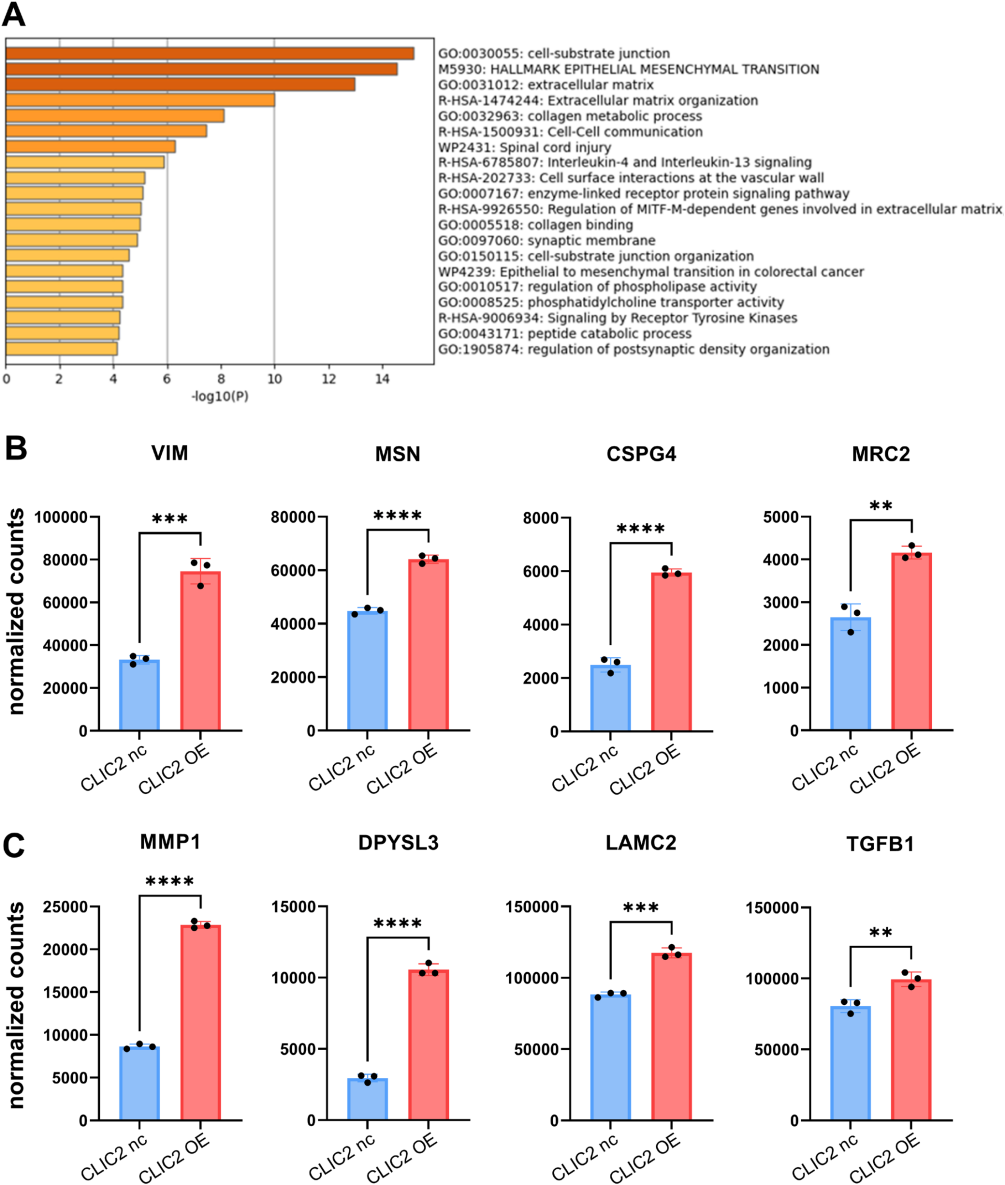
Analysis of upregulated genes in association with CLIC2 overexpression. (A) Metascape analysis was performed on the subset of genes from Figure 4B that were upregulated in response to CLIC2 overexpression. (B) Examples of differentially expressed genes identified from normalized count data within the gene set “GO:0030055 cell‐substrate junction.” (C) Examples of differentially expressed genes identified from normalized count data within the gene set “M5930 HALLMARK EPITHELIAL MESENCHYMAL TRANSITION.” **p < 0.01, ***p < 0.001, ****p < 0.0001 (compared with CLIC2 nc cells). [Color figure can be viewed at wileyonlinelibrary.com]

The Metascape analysis of the upregulated genes was performed, and the results strongly suggested the contribution of factors related to the “cell‐substrate junction” (Figure 5A). Genes such as VIM, MSN, CSPG4, and MRC2 among this pathway, which are known to induce tumor‐promoting alterations when highly expressed in tumors, are shown as examples in Figure 5B [34, 35, 36, 37]. Figure 5C shows part of the “hallmark epithelial mesenchymal transition” gene set. Among the factors shown in Figure 5C as examples, MMP1, LAMC2, and TGF‐β show large numbers of counts in RNA sequencing, implicating their possible role as mediators of CLIC2 OE effects on cells.

### 3.6 Exploration of Cellular Regulatory Pathways in Response to CLIC2 OE, Related to Downregulated Genes

**FIGURE 6:**
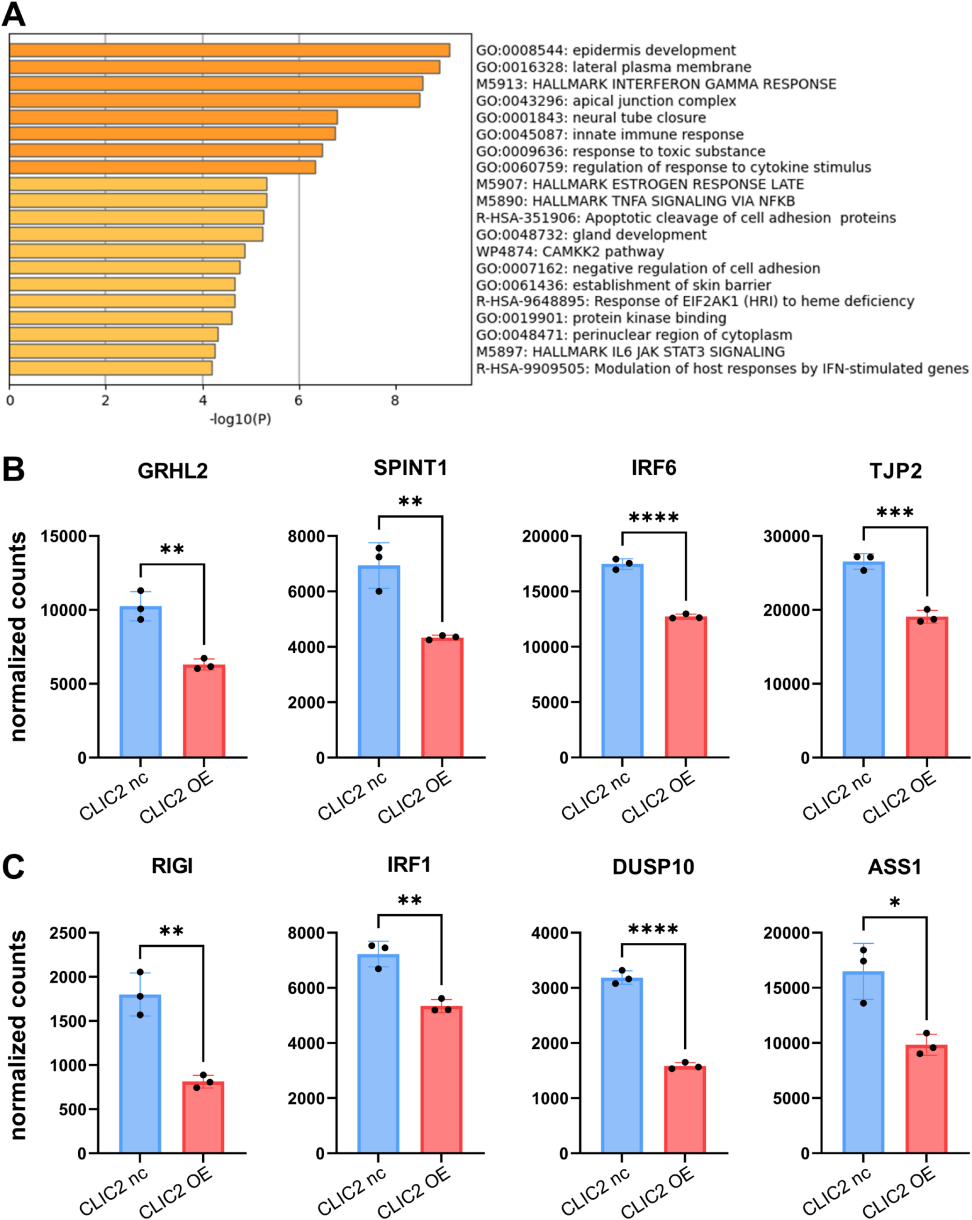
Analysis of downregulated genes in association with CLIC2 overexpression. (A) Metascape analysis was performed on a subset of genes from Figure 4B that were downregulated in response to CLIC2 overexpression. (B) Examples of differentially expressed genes identified from normalized count data within the gene set “GO:0008544 epidermis development.” (C) Examples of differentially expressed genes identified from normalized count data within the gene set “GO:0045087 innate immune response.” *p < 0.05, **p < 0.01, ***p < 0.001, ****p < 0.0001 (compared with CLIC2 nc cells). [Color figure can be viewed at wileyonlinelibrary.com]

The results of the Metascape analysis of the downregulated genes, overlapping with Figure 4, suggested a strong involvement of the “epidermis development” pathway (Figure 6A,C), implicating that changes in HNSCC traits as epithelial tissue changes related to the epidermal formation mechanism may be involved in CLIC2 OE's exacerbation. Considering the resistance of CLIC2 OE cells to tumor cell elimination by NK cells, genes associated with the “innate immune response” have attracted attention in the Metascape analysis of downregulated genes (Figure 6A). Among RIGI, IRF1, DUSP10, and ASS1 listed as representative genes in Figure 6B, RIGI is a receptor that detects viral infection and can trigger an immune response [38]. Furthermore, its downregulation may inhibit the mobilization of the immune response, potentially supporting the exacerbating effect of CLIC2 OE on the immune system.

### 3.7 Cases of CLIC2 Expression in Human HNSCC

**FIGURE 7:**
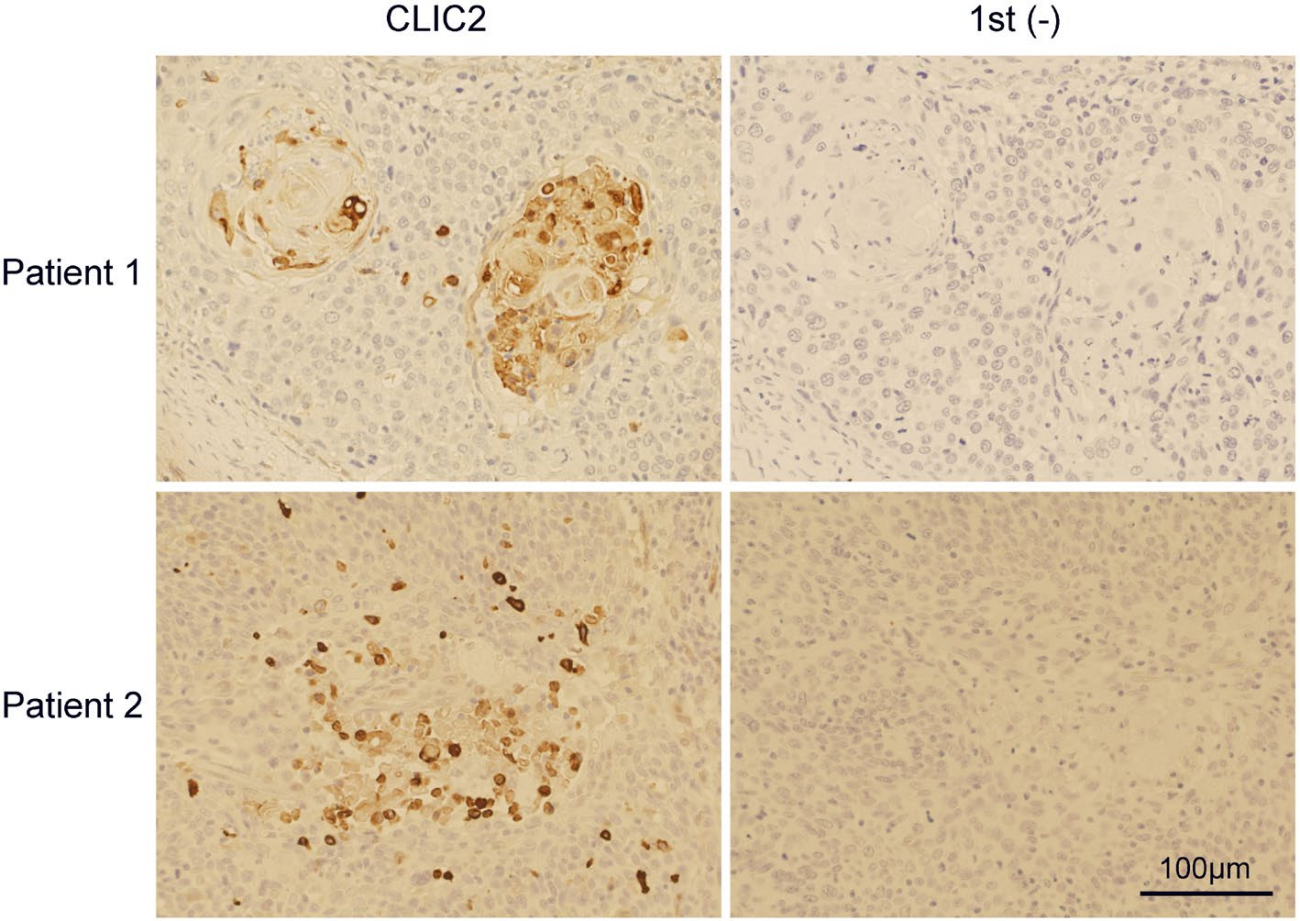
CLIC2 expression in human HNSCC. Immunohistochemistry was performed on serial sections of human HNSCC tissues. The target antigen was visualized in brown using an HRP–diaminobenzidine enzyme–substrate reaction. Counterstaining was performed with hematoxylin and eosin. Left column: Sections were incubated with an anti‐CLIC2 primary antibody. Right column: No primary antibody (negative control). Scale bar: 100 μm. [Color figure can be viewed at wileyonlinelibrary.com]

The results of the cultured cell study above suggest that CLIC2 can influence the malignancy of HNSCC cells, unlike in glioma cells. However, to the best of our knowledge, no studies have been conducted to determine whether such cases are possible in actual clinical practice. Therefore, we examined whether there were cases of CLIC2 expression in the clinical specimens of HNSCC collected at Ehime University Hospital (Figure 7) and summarized the results in Table 1. Histopathological examination of CLIC2 protein expression in tissues of patients with HNSCC revealed its expression in 7 of 15 cases (Table 1). Even in all CLIC2‐positive cases, not all tumor cells in the patient tissues expressed the CLIC2 protein; however, some cells were positive for CLIC2 expression, indicating heterogeneity of the tumor tissue and cells (Figure 7). The sample size is still insufficient for statistical analysis; however, the current trend at least suggests a tendency toward malignancy in CLIC2‐positive cases (Table 1).

## 4 Discussion

Current problems associated with chemotherapy for HNSCC include a high recurrence rate with strong adverse events and poor survival outcomes, especially in recurrence or metastasis [6]. ICIs have shown promising efficacy in previously uncontrolled cases; however, 60% of patients with recurrence or metastasis are reportedly refractory to ICIs [39, 40]. Nowadays, using CGP analysis, a comprehensive search for target genes is performed in patients with cancer, not limited to HNSCC, who are unresponsive to standard cancer treatments. However, only a few cases have succeeded in identifying therapeutic target genes for HNSCC by CGP and in obtaining a therapeutic response, as compared to that in other cancer types [9]. The inadequacy of current chemotherapy and the lack of benefit from CGP are major problems in treating HNSCC. Furthermore, finding a potential therapeutic target is an important and significant challenge. Consequently, we recently investigated the significance of the CLIC2 protein in HNSCC.

We investigated the significance of CLIC2 in HNSCC by establishing forced CLIC2‐expressing cells and analyzing their phenotypes. We found increased tumorigenicity in xenografts, enhanced proliferative potential in culture, resistance to tumor immunity, as well as increased expression of factors that we previously identified as possibly tumor‐promoting. These findings suggest that CLIC2 is tumor‐promoting in HNSCC, which contrasts with previous reports. Therefore, the significance of CLIC2 in tumors is cell context‐dependent, although no previous studies to our knowledge support or suggest this concept. This may be because of only limited studies on CLIC2. Nevertheless, another strong consideration is *CLIC2's* absence in the *Mus musculus* genome (https://www.informatics.jax.org/). The existence of *CLIC2* in other mammals including the closely related mouse, *Mus pahari* (https://www.informatics.jax.org/), demonstrates that the CLIC2 protein is not essential for life but is redundant. Moreover, the redundancy of CLIC2 may be the reason for its role being cell context dependent.

This cell context dependency can be confirmed in the effects of CLIC2 expression in different cellular lineages. Notably, when comparing the effect of CLIC2 expression on the gene expression profile of SASL1m cells (as shown in Figures 4, 5, 6) with that in glioma cells as previously reported (reanalyzed, shown in Supporting Information 1), the contents were clearly different. For example, in glioma cells, the expression of genes associated with cell–cell adhesion increases with CLIC2 expression, whereas in SASL1m, the expression of genes associated with cell‐substrate adhesion increases, which is a sharp contrast reminiscent of the epithelial–mesenchymal transition. These findings do not fully reveal the mechanisms by which CLIC2 contributes to cancer progression in a cellular context‐dependent manner; however, they are not inconsistent with the idea that they reflect such a mechanism. Further investigation on the mechanism by which CLIC2 functions adversely in the context of HNSCC cell gene expression profiles is on the way.

The absence of the *CLIC2* gene in *Mus musculus* can be a barrier to studies and reduce its popularity in basic biology. This may be a reason for the poor number of CLIC2 studies. Under these circumstances, this report can be valuable as it demonstrates, in combination with the clinical implications discussed below, why analysis of these “less noteworthy factors” should be performed.

Although gene expression profiling using mRNA sequencing was performed to examine the effects of CLIC2 expression on HNSCC comprehensively, this analysis was limited to exploring cellular changes downstream of CLIC2 expression, owing to the study design. On the spontaneous occurrence in the CLIC2‐positive cases mentioned above, we currently have no information on the cause and suggest future exploration. Nevertheless, we believe that the NGS/enrichment analysis performed in this study showed interesting gene expression changes, indicating that the ectopic expression of CLIC2 may have an exacerbating effect on HNSCC. Particularly, we focused on the relationship between NK cells and HNSCC since we found that the simultaneous knockdown of two factors, NHE1 and LOXL2, in HNSCC can promote elimination by NK cells [26]. Furthermore, we observed that tight junctions and ECM reorganization were closely related to this phenomena [26] (unpublished data). Considering that CLIC2 forced expression accompanies the opposite effects of the above example, that is, confers resistance to NK cells, analyzing the signaling changes that contribute to conferring this resistance would be insightful. Enrichment analysis revealed changes in signaling pathways related to innate immunity and interleukin signaling as well as cell adhesion and tissue formation. It is plausible that changes in HNSCC signals associated with the immune system affect the relationship with NK cells. Furthermore, the suggestion that changes in the extracellular microenvironment are affected is correlated with the previously mentioned findings [26]. Notably, an increase in the cell proliferative potential associated with CLIC2 expression was observed in the culture; however, enrichment analysis did not suggest changes in cell cycle‐related gene expression. This implies that CLIC2 expression does not contribute directly to cell cycle regulation; however, it may affect proliferative potential due to its effects on the extracellular microenvironment.

Finally, we sought to determine whether CLIC2 is expressed in human HNSCC and found 7 of 15 positive cases. This could be the first report of CLIC2 expression in HNSCC. A detailed examination of the staining images revealed positive signals in the intracellular as well as the region that appeared to be extracellular regions, which may reflect the secretion of the CLIC2 protein, as previously reported [16]. Analyses of glioma cases have shown that extracellularly distributed CLIC2 can regulate MMP activity [12]; however, it remains unclear whether this is also the case for HNSCC. Cases with strong CLIC2 positivity on histopathology may have a high malignant potential owing to CLIC2's tumor‐promoting effects in HNSCC. Indeed, the two CLIC2‐positive cases presented in Figure 7 were both advanced cancers that had already developed postoperative recurrence despite radical resection (Table 1). In addition, CLIC2‐positive staining was relatively frequently observed in patients with advanced cancer (Table 1), although statistical significance requires further examination of more cases. Thus, we propose that an investigation of additional cases of HNSCC is urgently required to determine the correlation among grade, prognosis, and CLIC2 expression.

## 5 Conclusion

We found that CLIC2, which has been suggested to be antitumor, can function in a tumor‐promoting effect and may need to be recognized as a risk factor for HNSCC. That tumor‐promoting property itself might be mediated by changes in immune responsiveness and reorganization of the extracellular microenvironment, although the molecular mechanisms leading to the expression of CLIC2 remain unclear.

## Funding

This work was supported by the Japan society for the promotion of science, Japan (grant number Y.H. 23K08988).

## Ethics Statement

All animal experiments were performed in accordance with the guidelines of the Ethics Committee for Animal Experimentation at Ehime University (approval number: 05‐U‐44‐16). The use of paraffin blocks prepared from HNSCC surgical specimens was approved by the Clinical Research Ethics Committee of Ehime University Hospital (approval number: 2309004).

## Conflicts of Interest

The authors declare no conflicts of interest.

## Supporting information


**Table S1:** Antibodies for Immunohistochemical staining.
**Table S2:** Primers for qPCR.

**Figure S1:** The enrichment analyses of the gene expression profile data of glioma cells associated with CLIC2 expression presented in *Neoplasia*, 2021 Aug; 23(8):754–765 were re‐performed using Metascape analyses. Enriched upregulated and downregulated pathways were presented, respectively.

## Data Availability

The data that support the findings of this study are available from the corresponding author upon reasonable request.
