# Supplementary material for: Chloride Intracellular Channel 2 Can Function as a Malignant Factor in Head and Neck Squamous Cell Carcinoma
Source: Head Neck. 2025 Dec 12;48(5):1259–71. doi: 10.1002/hed.70133 (PMC13055430; doi:10.1002/hed.70133)
Supplement: Supplementary file 1 — Table S1: Antibodies for Immunohistochemical staining. Table S2: Primers for qPCR. [file HED-48-1259-s002.docx]

Supplementary Table

Table S1

Antibodies for Immunohistochemical staining

| 1^st^ antibody | source | antibody | clone | concentration |
| --- | --- | --- | --- | --- |
| CLIC2 | abcam | Rabbit monoclonal | EPR6494 | 1/100 |

Antibodies for immunoblotting

| 1^st^ antibody | source | antibody | clone | concentration |
| --- | --- | --- | --- | --- |
| β-actin | Wako | Mouse monoclonal | --- | 1/10000 |
| CLIC2 | abcam | Rabbit monoclonal | EPR6494 | 1/2000 |
| LOXL2 | abcam | Rabbit polyclonal | NP_002309 | 1/1000 |
| NHE1 | BD Transduction | Mouse monoclonal | 54/NHE-1 | 1/10000 |
| PD-L1 | abcam | Rabbit monoclonal | 28-8 | 1/2000 |

Table S2

Primers for qPCR

| qPCR Primers | Forward | Reverse |
| --- | --- | --- |
| Human GAPDH | 5`- GTGGTCTCCTCTGACTTCAAC -3` | 5`- CCTGTTGCTGTAGCCAAATTC -3` |
| Human CLIC2 | 5`- TGTAAAGGCTGGAAGTGATGG -3` | 5`- AGGCTTTCTGGTCATGTCAAC -3` |
| Human CLIC4 | 5`- GAAGTCTTATGCCCTCCCAAG -3` | 5`- CCCTCTCCAGTGCTTCATTAG -3` |
| Human MMP1 | 5`- CTCTGACATTCACCAAGGTCTC -3` | 5`- GATTTCCTCCAGGTCCATCAAA -3` |
| Human MMP2 | 5`- GGCACCCATTTACACCTACA -3` | 5`- CCAAGGTCAATGTCAGGAGAG -3` |
| Human MMP9 | 5`- GGGCTTAGATCATTCCTCAGTG -3` | 5`- GCCATTCACGTCGTCCTTAT -3` |
| Human MMP14 | 5`- GCCCAATGGAAAGACCTACTT -3` | 5`- CCCTTCCCAGACTTTGATGTT -3` |
