# Supplementary material for: Chloride Intracellular Channel 2 Can Function as a Malignant Factor in Head and Neck Squamous Cell Carcinoma
Source: Head Neck. 2025 Dec 12;48(5):1259–71. doi: 10.1002/hed.70133 (PMC13055430; doi:10.1002/hed.70133)
Supplement: Supplementary file 2 — Figure S1: The enrichment analyses of the gene expression profile data of glioma cells associated with CLIC2 expression presented in Neoplasia, 2021 Aug; 23(8):754–765 were re‐performed using Metascape analyses. Enriched upregulated and downregulated pathways were presented, respectively. [file HED-48-1259-s001.docx]

**
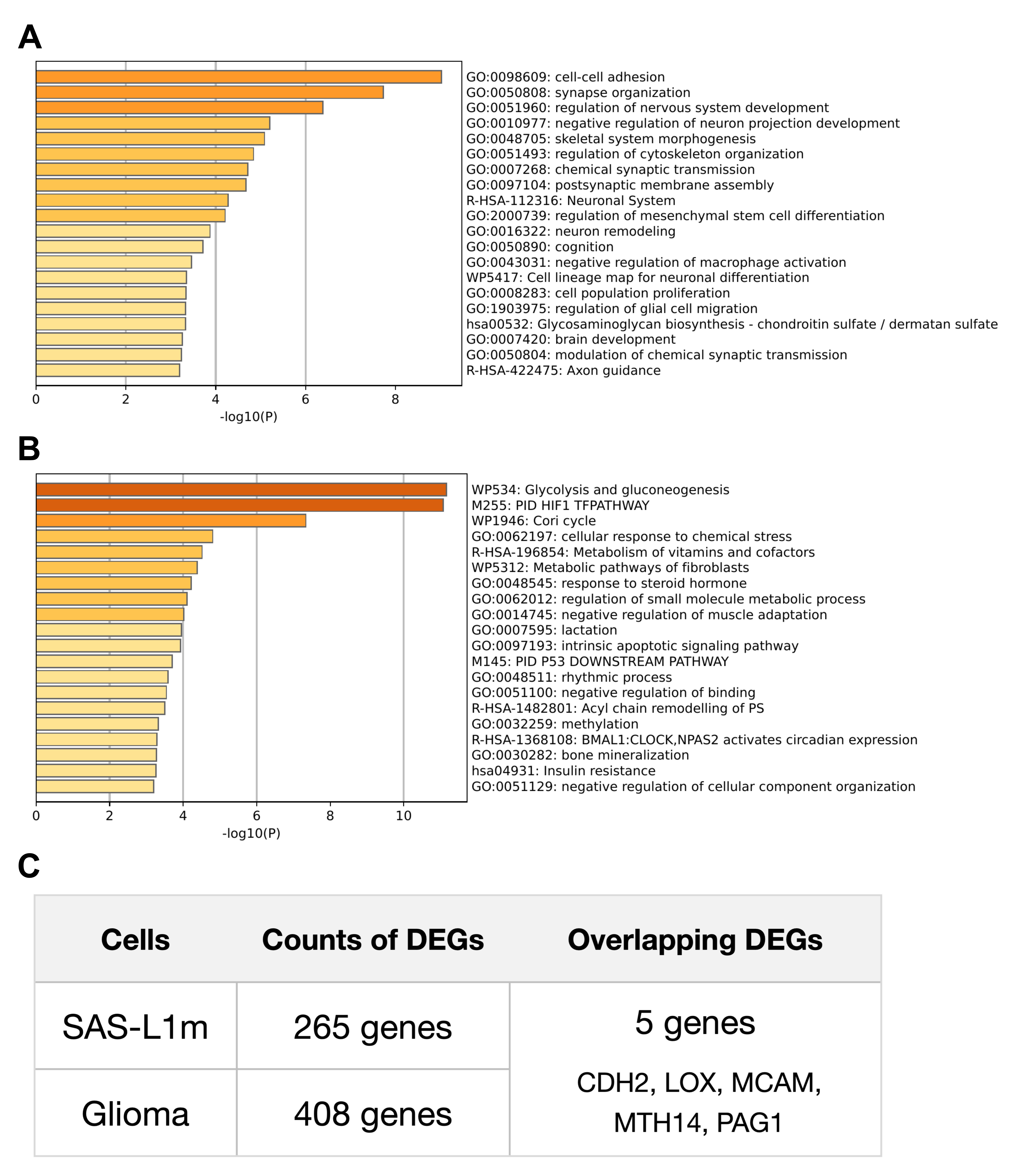
**

**Supplementary Figure**

The enrichment analyses of the gene expression profile data of glioma cells associated with CLIC2 expression presented in Neoplasia. 2021 Aug;23(8):754-765 were re-performed using Metascape analyses. Enriched upregulated and downregulated pathways were presented respectively.
